# Estimation of Cerebral Hemodynamics and Oxygenation During Various Intensities of Rowing Exercise: An NIRS Study

**DOI:** 10.3389/fphys.2022.828357

**Published:** 2022-03-02

**Authors:** Mikio Hiura, Yusuke Shirai, Hirohide Shibutani, Akio Funaki, Katsumi Takahashi, Yoichi Katayama

**Affiliations:** ^1^Center for Brain and Health Sciences, Aomori University, Aomori, Japan; ^2^Department of Sport and Health Science, Tokai Gakuen University, Miyoshi, Japan; ^3^Faculty of Sociology, Aomori University, Aomori, Japan; ^4^Faculty of Creative Engineering, Kanagawa Institute of Technology, Atsugi, Japan

**Keywords:** prefrontal cortex (PFC), cerebral blood volume (CBV), cerebral blood flow (CBF), cerebral metabolic rate for oxygen (CMRO_2_), effort, exhaustion, central fatigue, training

## Abstract

**Purpose:**

This study aimed to investigate changes in cerebral hemodynamics and oxygenation at moderate, heavy, maximal and supramaximal intensities of rowing exercise. It also examined whether these changes reflect alterations in sensation of effort and mood. We also aimed to examine the effects of peak pulmonary oxygen consumption ($\dot{V}$O_2p*eak*_) on cerebral oxygenation.

**Methods:**

Eleven rowers, consisting out of six athletes and five recreational rowers [two female; age, 27 ± 9 years; height, 171 ± 7 cm, body mass, 67 ± 9 kg; $\dot{V}$O_2p*eak*_, 53.5 ± 6.5 mL min^–1^ kg^–1^] rowed a 13-min session separated by 10 and 3 min, at 70 (Ex_70%_) and 80% of $\dot{V}$O_2p*eak*_ (Ex_80%_), respectively, on a rowing ergometer, followed by three sessions of 1-min supramaximal exercise (ExSp). After a warm-up at 60% of $\dot{V}$O_2p*eak*_ (ExM), seven male rowers performed a 2,000 m all-out test (Ex_2000_). Cardiovascular and respiratory variables were measured. Cerebral oxygenation was investigated by near-infrared time-resolved spectroscopy (TRS) to measure cerebral hemoglobin oxygen saturation (ScO_2_) and total hemoglobin concentration ([HbT]) in the prefrontal cortex (PFC) quantitatively. We estimated the relative changes from rest in cerebral metabolic rate for oxygen (rCMRO_2_) using TRS at all intensities. During Ex_70%_ and Ex_80%_, ratings of perceived exertion (RPE) were monitored, and alteration of the subject’s mood was evaluated using a questionnaire of Positive-and-Negative-Affect-Schedule after Ex_70%_ and Ex_80%_.

**Results:**

When exercise intensity changed from Ex_70%_ to Ex_80%_, the sense of effort increased while ScO_2_ decreased. [HbT] remained unchanged. After Ex_70%_ and Ex_80%_, a negative mood state was less prominent compared to rest and was accompanied by increases in both ScO_2_ and [HbT]. At termination of Ex_2000_, ScO_2_ decreased by 23% compared to rest. Changes in ScO_2_ correlated with $\dot{V}$O_2p*eak*_ only during Ex_2000_ (*r* = −0.86; *p* = 0.01). rCMRO_2_ did not decrease at any intensities.

**Conclusion:**

Our results suggest that alterations in the sense of effort are associated with oxygenation in the PFC, while positive changes in mood status are associated with cerebral perfusion and oxygen metabolism estimated by TRS. At exhaustion, the cerebral metabolic rate for oxygen is maintained despite a decrease in ScO_2_.

## Introduction

Rowing involves the large muscles and in total a high muscle mass of the entire body. Both strength and endurance are mandatory for achieving optimal competitive performance. In competitive rowing, the high-intensity work rate requires high metabolic demand, and all metabolic pathways are mobilized. In addition to the development of muscle hypertrophy with predominantly slow-twitch (or type I) fibers and the notable maximal oxygen uptake ($\dot{V}$O_2*max*_) (Larsson and Forsberg, 1980; Secher, 1993), elite rowers become exhausted because of extremely low levels of blood pH and arterial hypoxemia at the end of the competition (Nielsen et al., 1998, 1999). It has been suggested that the ability of the central nervous system to recruit motoneurons becomes limited during maximal-intensity rowing exercise, a limitation in the ability of the central nervous system to recruit motoneurons has been suggested (Roth et al., 1993; Volianitis et al., 2020). This limitation in recruiting slow-twitch fibers has been supposed to be due to the central fatigue mechanism (Secher et al., 2008; Taylor et al., 2016). Because of extreme fatigue induced by competitive rowing, alterations in cerebral blood flow (CBF) and oxygen metabolism, including the cerebral metabolic rate for oxygen (CMRO_2_), have been explored from the perspective of central fatigue (Nielsen et al., 1999; Volianitis et al., 2020). Previous studies have investigated the association between fatigue or exhaustion induced by maximal-intensity rowing exercise by measuring oxygenation of the prefrontal cortex (PFC) using near-infrared spectroscopy (NIRS) and the cerebral metabolic ratio, which is the oxygen-to-glucose index determined by arterial-to-internal jugular venous differences in the entire brain (Secher et al., 2008; Volianitis et al., 2020). Because of its practical utility, a large number of studies focused on oxygenation changes in the PFC during exercise (Herold et al., 2018; De Wachter et al., 2021). Furthermore, several studies using NIRS have investigated oxygenation in the PFC during rowing exercise (Nielsen et al., 1999, 2001; Faull et al., 2015). Although the PFC may not directly contribute to the neuronal control of movement, it is associated with various features of affective processing (Davidson, 2003), cognitive function (Fernandes et al., 2018), and mood status (Monroe et al., 2020) evoked by exercise. Cerebral oxygenation decreased before motor performance failure when exhaustion was elicited by maximal cycling exercise (Rupp and Perrey, 2008). Accordingly, the sense of effort and exhaustion evoked by high-intensity rowing exercise was reflected in the oxygenation in the PFC.

During training sessions for rowers, high-intensity training and repetitive sessions involving supramaximal intensity are mandatory (Treff et al., 2017); however, endurance training is often performed at low to moderate intensity (Nybo et al., 2014). When selecting exercise intensity during training sessions, the ratings of perceived exertion (RPE) and feelings experienced during exercise sessions are crucial indicators, because they are mirrored by physiological variables such as the heart rate (HR) and pulmonary oxygen consumption ($\dot{V}$O_2_), especially during steady-state continuous exercise. In addition to recognizing fatigue, it is important to know how rowers feel at different training intensities when examining these changes in sensations accompanying changes in the regional CBF and CMRO_2_. Because positron emission tomography (PET) cannot be used to measure regional CBF and/or CMRO_2_ during rowing ergometer exercise, we used NIRS to investigate cerebral oxygenation during rowing exercise and to estimate CMRO_2_, according to other methods involving NIRS (Brown et al., 2003; Roche-Labarbe et al., 2010).

Recently, near-infrared time-resolved spectroscopy (TRS) has enabled the in-depth measurement of absolute values of cerebral tissue oxygenation (Ohmae et al., 2006; Torricelli et al., 2014; Auger et al., 2016). Although the continuous-wave NIRS system is conventional and commercially available, it can only detect changes in oxygenated- and deoxygenated-hemoglobin concentrations in cerebral tissue because it is based on the modified Beer-Lambert law. TRS can continuously and simultaneously measure the absolute values of cerebral hemoglobin oxygen saturation (ScO_2_) and cerebral blood volume (CBV). ScO_2_ can be calculated by the ratio of oxyhemoglobin ([HbO]) and total hemoglobin ([HbT]) concentrations in the brain tissue, where [HbT] is the sum of [HbO] and deoxyhemoglobin concentration in the brain tissue ([HbR]) (Ijichi et al., 2005; Roche-Labarbe et al., 2010). Using [HbT], CBV is calculated using the molecular weight of hemoglobin, brain tissue density, and hemoglobin concentration of blood (HGB) (Kretschmann et al., 1986; Roche-Labarbe et al., 2010). Furthermore, we explored changes in CMRO_2_, not absolute values, by estimating the relationship between CBF and CBV reported by previous studies using PET and NIRS (Takahashi et al., 1999; Brown et al., 2003; Roche-Labarbe et al., 2010).

With a notable workload and maximal effort for competition, the physiological aspect of rowing demonstrates a unique challenge to the human capacity, including cerebral perfusion and metabolism (Volianitis and Secher, 2009). However, little is known about altered sensations of effort and fatigue caused by rowing exercise and brain metabolism associations. Therefore, this study aimed to investigate changes in oxygenation in the PFC during various exercise intensities used during regular training sessions for rowing in conjunction with cardiovascular and respiratory variables. To address this issue, we observed a session of rowing exercise consisting of two different intensities of constant-load exercise, that is, 70% (Ex_70%_) and 80% of $\dot{V}$O_2p*eak*_ (Ex_80%_), which were related to the heavy-intensity domain between the first (VT1) and the second ventilatory threshold (VT2) (Cerezuela-Espejo et al., 2018; Jamnick et al., 2020). Considering the actual rowing training session, supramaximal exercise (ExSp), which surpasses peak $\dot{V}$O_2_ ($\dot{V}$O_2p*eak*_) for a short duration, was also observed. Furthermore, we studied maximal exercise represented by the 2,000-m competition simulation (Ex_2000_) followed by moderate-intensity exercise (ExM), 60% of $\dot{V}$O_2p*eak*_, which corresponds to the intensity below VT1. To investigate the impact of the oxidative capacity on the extent of potential alterations in cerebral oxygenation, we investigated both moderately and highly trained rowers.

## Materials and Methods

### Participants

Eleven club-level rowers (two females, median age, 23 years; age range, 20–45 years) participated in this study. Characteristics of participants are shown in Table 1. Six male rowers were fit and performed competitive training of more than 6 h per week. Five rowers had retired or suspended their involvement in national-level competitions but continued to participate in recreational-level competitions and performed physical training fewer than 2 h per week. They had 2–23 years of rowing training and were familiar with Concept2 rowing ergometer (Concept2, Morrisville, United States) training and maximal rowing. None of the participants had a personal history of physical or psychiatric illness or substance abuse, and none was using any medications. The participants were advised to maintain an appropriate diet including carbohydrates and to stay well-hydrated. Additionally, they were instructed to avoid rigorous exercise, alcohol, and drugs during the 24 h preceding the experiments. All participants provided written informed consent after a detailed explanation of the study. The study protocol was designed in accordance with the guidelines of the national government and the 2008 revision of the Declaration of Helsinki. The protocol was approved by the Ethics Committee of the Faculty of Sociology, Aomori University (no. 03-2021).

**TABLE 1:** Demographic, physiological characteristics of the participants.

Participant ID	Performance level	Sex	Age (years)	Height (cm)	Body mass (kg)	Rowing experience (years)	Involvement in 2,000 m all-out test
1	C	M	20	179	65	2	+
2	C	M	22	171	70	4	+
3	C	M	23	173	65	7	+
4	C	M	22	180	74	7	
5	C	M	33	172	72	16	+
6	C	M	22	170	75	6	+
7	R	W	22	156	47	4	
8	R	W	23	164	56	5	
9	R	M	24	170	66	5	
10	R	M	45	170	68	23	+
11	R	M	44	180	80	23	+
Median (Total, *n* = 11)			23	171	68	6	
Median (competitive rowers, *n* = 6)			24	173	71	5	
Median (recreational rowers, *n* = 5)			22	170	66	5	

*C, competitive rowers; R, recreational rowers; W, women; M, men; +, participant who participated in 2,000-m maximal rowing.*

### Experimental Design

**FIGURE 1:**
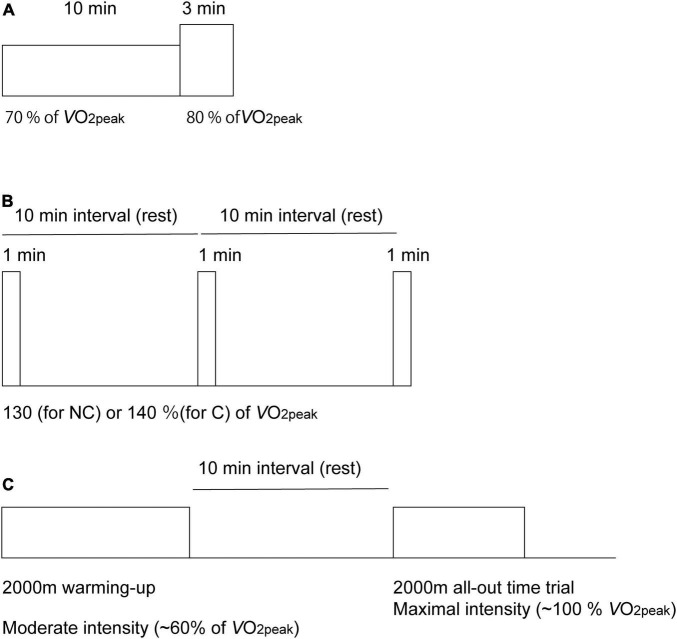
Diagram of the experimental protocol used for the acquisition of cerebral oxygenation using near-infrared time-resolved spectroscopy during the dual-intensities of constant-load exercise consisting of 10 min of rowing exercise at 70% of peak oxygen consumption ($\dot{V}$O_2p*eak*_), followed by 3-min of rowing exercise at 80% of $\dot{V}$O_2p*eak*_ **(A)**, three sessions of 1-min of supramaximal-intensity rowing exercise **(B)** and a 2,000-m maximal row (all-out time trial) simulating an on-water competition after a 2,000-m warm-up session **(C)**. Exercise intensities are normalized as the percentages of peak pulmonary oxygen consumption ($\dot{V}$O_2p*eak*_). During supramaximal intensity **(B)**, intensities were determined as 130% $\dot{V}$O_2p*eak*_ for the non-competing rowers (NC) and as 140% $\dot{V}$O_2p*eak*_ for the competitive rowers **(C)** group.

All participants completed an incremental test using the Concept2 ergometer until volitional exhaustion before the main experimental sessions. Within the 2 weeks after the incremental tests, the participants performed two experimental trials within a range of 4 weeks. The study protocol consisted of four sessions with five variations in exercise intensity and duration (Figure 1). During the first trial, participants performed a total of 13 min of exercise on a rowing ergometer at two different exercise intensities. After a 20-min period of recovery, they started three bouts of 1-min ExSp. On the second trial, seven male participants (five competitive and two recreational rowers) performed an Ex_2000_ simulating an on-water competition following a 2,000-m warmup session at 60% of $\dot{V}$O_2p*eak*_. During the experimental protocol, the HR and respiratory variables were measured. NIRS signals were explored on the forehead to investigate oxygenation in the PFC throughout the four experimental sessions. During each session, measurements were initiated 2 min before the start of exercise while the participants rested in the exercising position on the ergometer. At the dual-intensities of constant load exercise, the RPE (6–20 scale) (Borg, 1970) and mood alterations were evaluated using a questionnaire (Positive and Negative Affect Schedule (PANAS)] (Watson et al., 1988).

### Rowing Ergometer Incremental Test

As previously described (Jensen et al., 2021), seven 2-min incremental step tests were performed continuously without a break on a wind resistance-braked rowing ergometer (Model D, Concept2, Morrisville, United States) to determine $\dot{V}$O_2p*eak*_, and target power output (PO) for each experimental session. Before exercise started, rowers were equipped with the instruments and sat quietly for 1 min on the rowing ergometer before starting the exercise. The Concept2 rowing ergometer was equipped with a PM5-monitor, which allows the calculation of the PO averaged over 1 min. Respiratory variables were measured using an online gas analyzer (Quark CPET; Cosmed, Rome, Italy) in the breath-by-breath mode, and $\dot{V}$O_2_, CO_2_ production ($\dot{V}$CO_2_), minute ventilation ($\dot{V}$_*E*_), breathing frequency (R_*f*_), end-tidal CO_2_ (P_*ET*_CO_2_), and respiratory exchange ratio (RER) were continuously measured. HR was recorded using a Sport tester Polar 725X (Polar Electro Oy, Kempele, Finland). All data were averaged at 15-s intervals. $\dot{V}$O_2*peak*_ was defined as the maximum 15-s average $\dot{V}$O_2_. The maximal PO (MPO) was determined as the average power at the last completed step plus 25% of the starting step multiplied by the percentage of the 2 min completed during the last step (Jensen et al., 2021). The linear relationship between PO and $\dot{V}$O_2_ was determined and used to identify the target PO during different exercise intensities for the protocol. The first and second ventilatory thresholds (VT1 and VT2, respectively) were determined by a visual analysis. The VT1 was determined using the following criteria: the first increase in both the ventilatory equivalent of oxygen ($\dot{V}$_*E*_/$\dot{V}$O_2_) and end-tidal pressure of oxygen (P_*ET*_O_2_) with no concomitant increase in the ventilatory equivalent of carbon dioxide ($\dot{V}$_*E*_/$\dot{V}$CO_2_) during the incremental test. The VT2 was determined using the following criteria: the first increase in both the $\dot{V}$_*E*_/$\dot{V}$O_2_ and $\dot{V}$_*E*_/$\dot{V}$CO_2_ and a decrease in P_*ET*_CO_2_ (Cerezuela-Espejo et al., 2018).

### Main Experimental Sessions

#### Dual-Stage Constant-Load Rowing Exercise

The exercise session consisted of a constant load of 10-min of Ex_70%_, followed by a 3-min of Ex_80%_ (Figure 1A). PO for Ex_70%_ corresponded to 70% $\dot{V}$O_2p*eak*_, which was determined using the linear relationship between PO and $\dot{V}$O_2_ obtained during the incremental test described. Because VT1 was determined as 69 ± 6% using the data obtained data during the incremental test, 70% $\dot{V}$O_2p*eak*_ was determined to be the Ex_70%_. PO for Ex_80%_ corresponded to 80% $\dot{V}$O_2p*eak*_ because VT2 was determined to be 85 ± 5%. Cardiovascular and respiratory gas exchange variables and oxygenation in the PFC were measured throughout the exercise session. NIRS values and cardiorespiratory data were averaged at the last 15 s of rest, Ex_70%_, Ex_80%_ and 3 min after the exercise was terminated. To determine the cardiac output (CO), data were averaged at the last 30 s of rest, Ex_70%_, Ex_80%_ and 3 min after the exercise was terminated; fluctuations derived from body movement and respiration during rowing were considered. RPE was monitored at 10 min and 13 min after the exercise started. Ten minutes before exercise initiation and 3 min after the exercise was terminated, the mood of the participants was evaluated using the PANAS questionnaire.

#### Supramaximal Rowing Exercise

After 20 min of rest after the dual-staged constant-load rowing ergometer exercise, rowers performed three sessions of 1-min of ExSp, during which the intensity exceeded 130% $\dot{V}$O_2p*eak*_ for recreational rowers and 140% $\dot{V}$O_2p*eak*_ for competitive rowers (Figure 1B). ExSp started with a 10-min interval so that rowers had enough time to recover between the sessions. Cardiovascular and respiratory gas exchange variables and oxygenation in the PFC were measured throughout the exercise session. NIRS data and cardiorespiratory data were averaged at the last 15 s of rest before the exercise started as the baseline for each ExSp. Cardiorespiratory data were averaged at the last 15 s of the 1-min ExSp. NIRS data were collected when these values attained the nadir after the termination of the exercise.

#### 2,000 m All-Out Row Following a Warmup Session

Seven male rowers (five competitive and two recreational rowers) were included. The rowers performed their routine programs before the Ex_2000_ time trial. A 2,000-m warm-up rowing session, during which exercise intensity was targeted at approximately 60% $\dot{V}$O_2p*eak*_ (ExM), was included. This intensity corresponded to the level below VT1. After a 10-min rest, the rowers started Ex_2000_ and were encouraged to develop maximal effort. Cardiovascular variables, respiratory gas exchange variables, and oxygenation in the PFC were measured throughout the exercise session (during the warmup and all-out rows). NIRS values and cardiorespiratory data were averaged at the last 15 s of rest before the exercise started as the baseline and at the last 15 s of ExM and Ex_2000_ as the termination of exercise. Measurements of cardiovascular and respiratory gas exchange variables and NIRS values were continued until 2 min after ExM was terminated. NIRS measurements were continued until 4 min after Ex_2000_ was terminated, whereas measurements of cardiovascular and respiratory gas exchange variables were completed immediately after the exercise was terminated.

### Cardiovascular and Respiratory Gas Exchange Measurements

CO, systolic blood pressure (SBP), and diastolic blood pressure (DBP) were measured with an impedance cardiograph (Physio Flow Enduro, Manatec Biomedical, Paris, France) only for the dual-staged constant-load rowing exercise, Ex_70%_, and Ex_80%_. Measured data were averaged and indicated at 5-s intervals. As the diastolic period is reduced disproportionately more than the systolic period, mean blood pressure (MBP) is calculated as follows: MBP = DBP + Fs * (SBP–DBP), where Fs denotes the duration of arterial systole as a fraction of the cardiac cycle (Rogers and Oosthuyse, 2000). As Physio Flow Enduro provides the left ventricular ejection time (LVET) but not the pre-ejection period (PEP), it does not indicate the duration of arterial systole, which is the sum of LVET and PEP. Accordingly, we estimated MBP using the LVET as a fraction of the cardiac cycle. For the other two sessions including Ex_2000_ and ExSp, CO measurements were not applied because the signals were unstable because of artifacts introduced by body movement. HR was measured using the Sport tester Polar 725X (Polar Electro Oy, Kempele, Finland). Respiratory gas exchange variables were measured throughout all four sessions with the breath-by-breath mode using an online gas analyzer (Quark CPET, Cosmed) as described previously. All data were averaged at 15-s intervals. During the exercise sessions, except for Ex_2000_, measurements were begun 2 min before the exercise started and continued 5 min after the exercises were completed. During Ex_2000_, measurements of respiratory gas exchange measurements were stopped immediately after the session was completed.

### Near-Infrared Spectroscopy Measurements

We used a portable three-wavelength TRS system (tNIRS; Hamamatsu Photonics K.K., Hamamatsu, Japan) to quantitatively measure changes in PFC oxygenation. The TRS system uses a time-correlated single-photon counting technique for detection. The precise methodology has been previously described (Ijichi et al., 2005; Torricelli et al., 2014; Lange and Tachtsidis, 2019). Briefly, the system consists of three-picosecond light pulsers with different wavelengths (755, 816, and 850 nm), with a 100-ps duration at a repetition frequency of 5 MHz as the pulse light source, a photon-counting head for single-photon detection, and signal-processing circuits for time-resolved measurements. The light emission and detection optodes were positioned on the forehead just below the hairline with a 30-mm interoptode distance. Based on our previous study (Hiura et al., 2018), the location probes were allocated using the International EEG 10–20 system for electrode placement (Klem et al., 1999). The optodes were placed over the left and right sides of the forehead between Fp1 and F3, and between Fp2 and F4 to maximize the probability of photon transmission through the lateral portions of Brodmann areas 9 and 46 (Okamoto et al., 2004). The optodes were fixed with black-colored rubber to prevent stray light from reaching the detector. The covered optodes were firmly adhered to the skin with transparent tape. Furthermore, to prevent movement, the participant wore a cap with an opaque cloth attached. The photons passed through the scalp, skull, and frontal lobe to a depth of several centimeters, with only a minimal influence on skin blood flow. The TRS method provided absolute values of cerebral HbO and HbR. ScO_2_, HbT, and CBV were calculated as follows:


(1) $${\text{ScO}}_{2}\left(\%\right)=\frac{\text{[HbO]}}{\left[\text{HbO}\right]+\text{[HbR]}}$$

(2) [HbT]=[HbO]+[HbR]

(3) $$\text{CBV}\,\left(\text{mL}/100\,\text{g}\right)=\frac{\text{HbT}\times {\text{MW}}_{\text{Hb}}}{\text{HGB}\times {\text{D}}_{\text{bt}}}$$

where [HbO], [HbR], and [HbT] indicate the concentrations of oxyhemoglobin, deoxyhemoglobin and total hemoglobin in the brain tissue (μM), respectively, obtained using TRS, MW_*Hb*_ is the molecular weight of hemoglobin (64,500), HGB is the blood hemoglobin concentration (g/dL), and Dbt is the brain tissue density (1.05 g/mL) (Ijichi et al., 2005; Ishii et al., 2014).

To estimate relative CMRO_2_ (rCMRO_2_) using TRS, we used the following equation:


$${\text{CMRO}}_{2}=\text{CBF}\times 1.39\times \text{HGB}\times \left({\text{SaO}}_{2}-{\mathrm{SvO}}_{2}\right)$$

where SaO_2_ is arterial hemoglobin and SvO_2_ is venous hemoglobin.

ScO_2_ was calculated using the following equation (Watzman et al., 2000):


$${\text{ScO}}_{2}=\alpha \,{\text{SaO}}_{2}+\beta \,{\text{SvO}}_{2},\mathrm{with}\,\alpha +\beta =1$$

where α and β are constants.

Using these formulas and applying SaO_2_ and ScO_2_, we obtained the following:


$${\text{CMRO}}_{2}=\text{CBF}\times 1.39\times \text{HGB}\times \frac{{\text{SaO}}_{2}-{\text{ScO}}_{2}}{\beta }$$

Because we assumed a constant power-law relationship among changes in CBF and CBV (Roche-Labarbe et al., 2010) and considered the reference status (e.g., at rest), relative changes in CMRO_2_ (rCMRO_2_) were calculated as follows:


$${\text{rCMRO}}_{2}=\frac{{\text{CMRO}}_{2}}{{\text{CMRO}}_{2\,\mathrm{rest}}}=\frac{\text{HGB}}{{\text{HGB}}_{\text{rest}}}\times {\left(\frac{\text{CBV}}{{\text{CBV}}_{\text{rest}}}\right)}^{\gamma }\times \left(\frac{{\text{SaO}}_{2}-\mathrm{Sc}\,{\text{O}}_{2}}{{\text{SaO}}_{2\,\mathrm{rest}}-\mathrm{Sc}\,{\text{O}}_{2\,\mathrm{rest}}}\right)$$

with γ = 2.6 (Grubb et al., 1974; Brown et al., 2003) and the subscript “rest” indicated the baseline values.

Because arterial and venous blood samplings were not available in the present study, we assumed that changes in HGB and SaO_2_ would be identical to the results of previous studies with similar exercise protocol (Nielsen et al., 1998; Nielsen, 2003; González-Alonso et al., 2004). We assumed that HGB would increase by 5, 6, 7, and 10% with ExM, Ex_70%_, Ex_80%_, and Ex_2000_, respectively, while the influence of the short duration of ExSp would be negligible. We assumed SaO_2_ to be 98% for the baseline; however, we assumed it to be 97, 96, 95, and 92% for ExM, Ex_70%_, Ex_80%_, and Ex_2000_, respectively. For ExSp, SaO_2_ was assumed to be 97%. Although the HGB values for five participants were examined within 1 week of the main study, we referred to these values only to clarify whether [HbT] was attributed as an outlier. As shown in the aforementioned formula, individual HGB was not necessary to estimate rCMRO_2_ and relative changes in CBV (i.e., the ratio of CBV to CBV_*rest*_ [rCBV]).

To evaluate the effect of different types of exercise intensities on cerebral oxygenation and hemodynamics, relative changes in ScO_2_ (ΔScO_2_) and [HbT] (ΔHbT), rCMRO_2_, and rCBV were measured at the end of exercise and at 2–4 min after exercise termination. Values obtained at the last 15 s of exercise were defined as the effect at the end of exercise and compared with those at rest for all sessions. Values obtained at 3 min after the exercise was terminated were used as the effect after exercise termination for Ex_70%_, Ex_80%_ and ExSp and compared with those at rest. For the dual-stage constant-load rowing exercise, the effect of at the end of Ex_80%_ was regarded as the sum of Ex_70%_ and Ex_80%_ (Ex_70%_ + Ex_80%_). For ExM and Ex_2000_, values obtained at approximately 2 and 4 min after the exercise was terminated were used, respectively, as the effect after exercise termination compared with those at rest. These time points corresponded to the 20th and 50th percentiles of the total elapsed time of ExM and Ex_2000_, respectively, considering the inter-individual differences.

When significant changes in ScO_2_ or [HbT] for any exercise session were identified, we examined whether ΔScO_2_ and ΔHbT were correlated with relative $\dot{V}$O_2p*eak*_, $\dot{V}$O_2p*eak*_, and MPO.

### Statistics

Data were analyzed using GraphPad Prism 9 (GraphPad Software, Inc, San Diego, CA, United States) and SPSS version 25 (IBM, Armonk, NY, United States). The average data are expressed as the arithmetic mean ± standard deviation, unless otherwise stated. Normal Gaussian distribution of normality was performed using the Shapiro-Wilk test. To calculate differences between groups, a *t*-test was performed after testing the normality of distribution using the Shapiro-Wilk test. To evaluate differences between time points for the examined variables throughout each exercise session, a one-way repeated-measures analysis of variance (ANOVA) with Turkey’s honestly significant difference *post hoc* procedure was performed. For NIRS variables, one-way repeated-measures ANOVA with Tukey’s HSD *post hoc* procedure was performed to evaluate the difference between rest, during exercise, and after exercise. The magnitude of the difference was assessed by the effect sizes (Cohen’s d; d or partial eta squared; η^2^) and defined as small (≥0.2 to < 0.5), medium (≥0.5 to <0.8), and large (≥0.8) for d, and small (≥0.01 to < 0.06), medium (≥ 0.06 to < 0.14), and large (≥0.14) for η^2^ (Cohen, 1988). Pearson’s correlation coefficients were used to assess relationships between two variables of interest.

## Results

As training years, status, body size, age and sex differed among rowers, $\dot{V}$O_2p*eak*_ and relative $\dot{V}$O_2p*eak*_ ranged from 2.3 to 4.8 L min^–1^ (3.6 ± 0.7) and from 43 to 65 mL min^–1^kg^–1^, respectively. Because the NIRS data of two rowers were not completely available throughout the exercise sessions because of artifacts probably caused by sweat and head movement, we applied data obtained from the left forehead for nine rowers and from the right forehead for two rowers. An evaluation of the NIRS data obtained from the right PFC of two rowers indicated similarities in the NIRS trends when both sides were available.

### Dual-Staged Constant-Load Rowing Exercise

**FIGURE 2:**
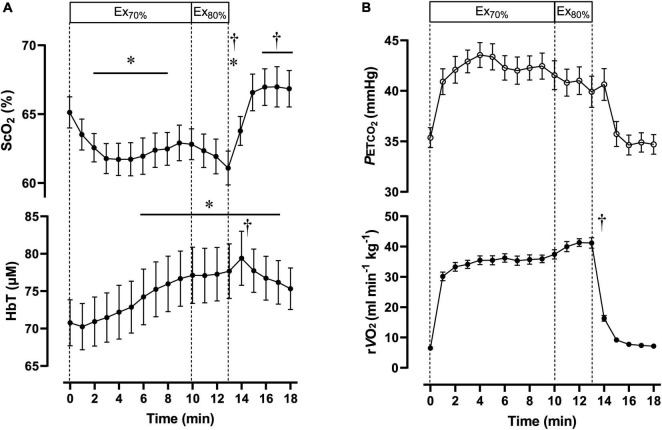
Cerebral oxygenation variables obtained using near-infrared spectroscopy for the prefrontal cortex **(A)** and respiratory variables **(B)** during the dual-stage constant-load rowing exercise for 10-min at 70% of peak oxygen consumption ($\dot{V}$O_2p*eak*_) followed by 3-min of rowing ergometer exercises at 80% of $\dot{V}$O_2p*eak*_. Solid lines indicate baseline (0 min) and at the end of Ex_70%_ (10 min) and Ex_80%_ (13 min) exercise. Data were averaged over 15 s for each time point. Ex_70%_, exercise at 70% of $\dot{V}$O_2p*eak*_; Ex_80%_, exercise at 80% of $\dot{V}$O_2p*eak*_; ScO_2_, cerebral hemoglobin oxygen saturation; HbT, total hemoglobin concentration in the brain tissue; r$\dot{V}$O_2_, relative pulmonary oxygen consumption; P_*ET*_CO_2_, end-tidal carbon dioxide. Significant difference compared to baseline; *P < 0.05. Significant difference compared to the end of exercise at 70% of $\dot{V}$O_2p*eak*_ (10 min); ^†^*P* < 0.05. Statistical differences between each timepoint were determined using a one-way analysis of variance for repeated measures and Turkey’s *post hoc* multiple comparisons test.

Table 2 contains a summary of cardiovascular and respiratory variables at the baseline, Ex_70%_, Ex_80%_, and 3 min after exercise termination. Cardiovascular and respiratory variables (except for HR, CO, $\dot{V}$_*E*_, $\dot{V}$CO_2_, and RER) increased at the end of Ex_70%_ and Ex_80%_ and returned to the baseline levels. A summary of NIRS variables during the session are shown in Table 3. The magnitude of changes in ScO_2_ and [HbT] were large compared with other NIRS variables. Changes in ScO_2_ and [HbT] throughout the exercise session are presented with those in P_*ET*_CO_2_ and relative $\dot{V}$O_2p*eak*_ in Figure 2.

**TABLE 2:** Cardiovascular and respiratory variables in response to 10-min of 70% $\dot{V}$O_2p*eak*_-intenity followed by 3-min of 80% $\dot{V}$O_2p*eak*_-intenity rowing ergometer exercises.

	Rest	Ex_70%_	Ex_80%_	Post-3 min
Heart rate (beats min^–1^)	78 ± 11	156 ± 14***	167 ± 15***,^††^	104 ± 13**
MBP (mmHg)	90 ± 6	102 ± 5***	107 ± 5***,^††^	90 ± 7
Cardiac output (l min^–1^)	5.8 ± 0.9	15.0 ± 2.6***	18.1 ± 3.3***,^††^	8.2 ± 1.1**
$\dot{V}$_*E*_ (l min^–1^)	13 ± 2	73 ± 20***	88 ± 21***,^††^	22 ± 7**
R_*f*_ (breaths min^–1^)	19 ± 4	45 ± 10***	49 ± 10***	24 ± 8
$\dot{V}$O_2_ (l min^–1^)	0.4 ± 0.1	2.5 ± 0.6***	2.9 ± 0.6***,^††^	0.5 ± 0.1
Relative $\dot{V}$O_2_ (ml min^–1^kg^–1^)	6.5 ± 1.4	37.4 ± 5.1***	41.2 ± 5.7^†^	7.7 ± 1.7
P_*ET*_CO_2_ (mmHg)	35 ± 3.0	42 ± 4.8**	40 ± 5.1*	36 ± 4.2
$\dot{V}$CO_2_ (l min^–1^)	0.4 ± 0.1	2.5 ± 0.6***	2.9 ± 0.7***,^††^	0.6 ± 0.2**
RER	0.84 ± 0.07	0.99 ± 0.03**	1.06 ± 0.03***,^††^	1.20 ± 0.07***

Values are expressed as mean ± standard deviation. (N = 11) at baseline (Rest), at the end of 10-min of constant-load exercise at 70% of peak oxygen consumption ($\dot{V}$O_2peak_; Ex_70%_), and during 3-min of constant-load exercise at 80%$\dot{V}$O_2peak_ (Ex_80%_), and at 3 min after exercise termination (Post-3 min). Data were averaged over 15 s for each time point. MBP, mean blood pressure;$\dot{V}$_E_, minute ventilation; R_f_, breathing frequency;$\dot{V}$O_2_, pulmonary oxygen consumption; P_ET_CO_2_, end-tidal carbon dioxide,$\dot{V}$CO_2_, CO_2_ production; RER, respiratory exchange ratio. Significant difference compared to rest: *P < 0.05, **P < 0.01, ***P < 0.001. Significant difference compared to Ex_70%_: ^†^P < 0.05, ^††^P < 0.001.

**TABLE 3:** Changes in near-infrared spectroscopy signals in response to 10-min of 70% $\dot{V}$O_2p*eak*_-intenity followed by 3-min of 80% $\dot{V}$O_2p*eak*_-intenity rowing ergometer exercises.

	Rest	Ex_70%_	ExH_80%_	Post-3 min	Effect sizes (η^2^-partial)
ScO_2_ (%)	64.4 ± 4.0	62.0 ± 3.8	60.2 ± 4.0**,^††^	66.5 ± 4.3*, ^††,§§^	0.872
HbT (μM)	70.4 ± 9.3	76.5 ± 11.2**	77.5 ± 11.7**	78.9 ± 11.4**, ^††,§§^	0.881
O_2_Hb (μM)	43.1 ± 8.8	45.4 ± 9.6	44.9 ± 9.1	49.5 ± 11.5*,^†,§^	0.576
HHb (μM)	25.1 ± 4.4	29.2 ± 4.9**	30.9 ± 5.7**,^††^	26.6 ± 5.1^††,§§^	0.803

Values are expressed as mean ± standard deviation. (N = 11) at baseline (Rest), at the end of 10-min of constant-load exercise at 70% of peak oxygen consumption ($\dot{V}$O_2peak_; Ex_70%_), during 3-min of constant-load exercise at 80%$\dot{V}$O_2peak_ (Ex_80%_), and at 3 min after exercise termination (Post-3 min). Data were averaged over 15 s for each time point. ScO_2_, cerebral hemoglobin oxygen saturation; HbT, total hemoglobin concentration in the brain tissue; HBO, oxyhemoglobin concentration in the brain tissue; HHb, deoxyhemoglobin concentration in the brain tissue. Significant difference compared to rest: *P < 0.05, **P < 0.01. Significant difference compared to Ex_70%_: ^†^P < 0.05, ^††^P < 0.01. Significant difference compared to Ex_80%_: ^§^ P < 0.05, ^§§^ P < 0.01.

During Ex_70%_, the PO was 147 ± 34 W, corresponding to 56 ± 5% MPO and relative $\dot{V}$O_2_ increased to 67 ± 6 of $\dot{V}$O_2p*eak*_. RPE recorded at 10 min during the exercise session was 12 ± 1.6 arbitrary units (a.u.). [ScO_2_] began to decrease at the onset of Ex_70%_ and had significantly lower values than that at rest from 2 to 8 min when analyzed throughout the session. [HbT] gradually increased during Ex_70%_ and was significantly higher than that at rest after 6 min when the exercise was started.

During Ex_80%_, PO was 187 ± 48 W, corresponding to 70 ± 4% of MPO, and relative $\dot{V}$O_2_ increased to 79 ± 6 of $\dot{V}$O_2*peak*_, with further increases in CO and MBP compared with Ex_70%_. RPE increased to 13 ± 1.6 a.u. compared with the end of Ex_70%_ (*p* < 0.001). The manipulation of exercise intensity evoked significant increases in all cardiovascular and respiratory variables except for P_*ET*_CO_2_ at the end of Ex_80%_ compared with Ex_70%_. [ScO_2_] began to decrease again at the onset of Ex_70%_, and it reached its nadir at the end of Ex_80%_. [HbT] increased from that at rest during Ex_80%_, but it did not increase further compared with Ex_70%_.

After exercise termination, MBP, R_*f*_, $\dot{V}$O_2_, and P_*ET*_CO_2_ returned to the same level as those at rest, but other cardiovascular and respiratory variables increased compared to the values at rest values. [ScO_2_] sharply increased after exercise termination, attaining the highest value at 5 min after exercise termination. [ScO_2_] increased to 66.6 ± 4.0%, without a significant difference compared to that at rest. [HbT] had a peak of 79.4 ± 11.9 μM at 1 min after exercise termination; then, it gradually decreased, staying at a higher level than that at rest until 5 min after exercise termination. The PANAS negative score changed significantly from 17 ± 5.1, at rest, to 13 ± 5.6 a.u., after Ex_80%_ (*p* < 0.01), while the PANAS positive score did not change significantly (from 29 ± 4.9 to 30 ± 6.4 a.u.; *p* = 0.13).

### Supramaximal Rowing Exercise

**FIGURE 3:**
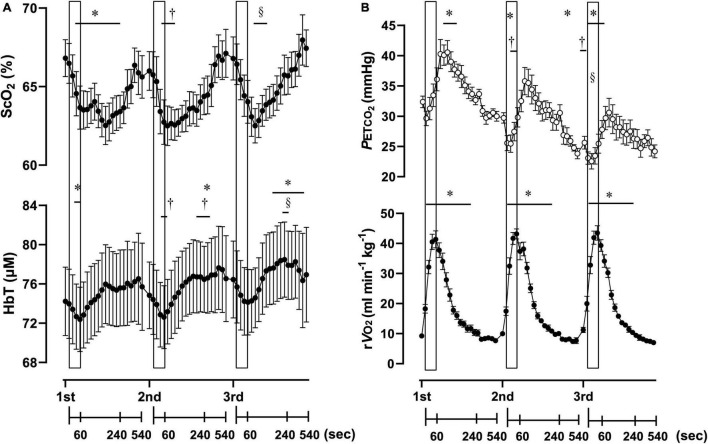
Cerebral oxygenation variables obtained using near-infrared spectroscopy for the prefrontal cortex **(A)** and respiratory variables **(B)** of three sessions of 1-min of supramaximal rowing exercise. Each exercise bout started with a 10-min interval. Data are plotted with samplings of 15 s for 4 min from the start, and then with samplings of 60 s from 5 to 9 min for graphical representation. Solid rectangles representing 1-min sessions are placed at the time when exercise was started. ScO_2_, cerebral hemoglobin oxygen saturation; HbT, total hemoglobin concentration in the brain tissue; r$\dot{V}$O_2_, relative pulmonary oxygen consumption; P_*ET*_CO_2_, end-tidal carbon dioxide. Significant difference compare to baseline of the first exercise session; *P < 0.05. Significant difference compared to baseline of second exercise session; ^†^*P* < 0.05. Significant difference compared to baseline of the third exercise session; ^§^ *P* < 0.05. Statistical differences between each timepoint were determined using a one-way analysis of variance for repeated measures and Turkey’s *post hoc* multiple comparisons test.

Three sessions of ExSp evoked volitional exhaustion during each session. For three repetitions, PO values were 359 ± 135 W, 357 ± 115 W, and 352 ± 116 W, corresponding to 132 ± 24, 133 ± 11, and 131 ± 13% of MPO, respectively. There was no significant difference in PO among the three sessions [*F*(2, 125) = 0.20, *p* = 0.70]. Table 4 contains a summary of cardiovascular and respiratory variables at the baseline and end of each bout. With 10-min intervals, the baseline values at each ExSp for HR, $\dot{V}$O_2_, and $\dot{V}$CO_2_ were identical, while respiratory variables were changed as sessions accumulated. During each ExSp session, the relative $\dot{V}$O_2_ increased to 81 ± 14, 84 ± 8, and 83 ± 10% of $\dot{V}$O_2p*eak*_, respectively. P_*ET*_CO_2_ significantly differed from baseline during the first and third sessions, and it decreased at the end of the second and third sessions compared with that of the first session. The trends of the variables measured using NIRS are shown in Table 5. Changes in [ScO_2_] and [HbT] throughout the three sessions are presented with those of P_*ET*_CO_2_ and relative $\dot{V}$O_2p*eak*_ in Figure 3. The baseline values at each ExSp for the NIRS samples were identical. [ScO_2_] continued to decrease during each session and reached its nadir at 105, 15, and 30 s after exercise termination. [HbT] had its nadir exactly at the termination of each session and significantly decreased only during the first and second sessions compared with the baseline values. The magnitude of changes in [ScO_2_] was larger than that of [HbT] throughout the session.

**TABLE 4:** Cardiovascular and ventilatory variables in response to three sessions of 1-min of supramaximal-intensity rowing exercise.

	First		Second		Third	
	Pre-Ex1	Ex1	Pre-Ex2	Ex2	Pre-Ex3	Ex3
Heart rate (beats min^–1^)	102 ± 16	164 ± 20	111 ± 13**	170 ± 12	116 ± 12***	170 ± 11
$\dot{V}$_*E*_ (breaths min^–1^)	22 ± 9	110 ± 22	28 ± 9	109 ± 26	32 ± 9**	119 ± 33*
R_*f*_ (breaths min^–1^)	21 ± 4	61 ± 16	25 ± 6	69 ± 15	27 ± 6**	75 ± 13^§^
$\dot{V}$O_2p*eak*_ (l min^–1^)	0.5 ± 0.2	2.9 ± 0.8	0.6 ± 0.1	3.0 ± 0.6	0.6 ± 0.2	3.0 ± 0.7
Relative $\dot{V}$O_2_ (ml min^–1^kg^–1^)	6 ± 0.8	43 ± 9	8 ± 1.3	45 ± 6	7 ± 1.4	44 ± 7
P_*ET*_CO_2_ (mmHg)	32 ± 3	36 ± 6	30 ± 3	29 ± 6^§§^	26 ± 3**, ^§§^	26 ± 5^§§^
$\dot{V}$CO_2_ (l min^–1^)	0.6 ± 0.2	3.1 ± 0.7	0.7 ± 0.2	2.6 ± 0.5^§§^	0.7 ± 0.2	2.6 ± 0.6^§^
RER	1.05 ± 0.13	1.01 ± 0.13	1.10 ± 0.13	0.91 ± 0.12	1.03 ± 0.14	0.91 ± 0.08

Values are expressed as mean ± standard deviation. (N = 11) at baseline of the first session (Pre-Ex1), the end of the first session (Ex1), at baseline of the second session (Pre-Ex2), at the end of the second session (Ex2), at baseline of the third session (Pre-Ex3), and the end of the third session (Ex3). Data were averaged over 15 s for each time point. V_E_, minute ventilation; R_f_, breathing frequency;$\dot{V}$O_2_, pulmonary oxygen consumption; P_ET_CO_2_, end-tidal carbon dioxide,$\dot{V}$CO_2_, carbon dioxide production; RER, respiratory exchange ratio. At Ex1, Ex2, and Ex3, all variables, except for P_ET_CO_2_ and RER significantly changed compared with the corresponding baselines (P < 0.01). Significant difference compared to Pre-Ex1 for Pre-Ex2 and/or Pre-Ex3: *P < 0.05, **P < 0.01, ***P < 0.001. Significant difference compared to Pre-Ex2 at Pre-Ex3: ^††^P < 0.01. Significant difference compared to Ex1 at Ex2 and/or Ex3: ^§^P < 0.05, ^§§^P < 0.01.

**TABLE 5:** Changes in near-infrared spectroscopy signals in response to three bouts of 1-min supramaximal-intensity rowing ergometer exercises.

	First			Second			Third		
	Pre-Ex1	Ex1	ES (d)	Pre-Ex2	Ex2	ES (d)	Pre-Ex3	Ex3	ES (d)
ScO_2_ (%)	66.8 ± 3.9	62.5 ± 3.9**	1.19	66.0 ± 4.0	62.5 ± 4.2**	0.89	66.8 ± 4.6	62.5 ± 3.6**	1.08
HbT (μM)	74.2 ± 11.6	72.4 ± 10.9**	0.17	74.8 ± 11.3	72.6 ± 10.6**	0.21	76.5 ± 13.0	74.1 ± 11.1	0.21
HbO (μM)	49.7 ± 9.6	45.8 ± 9.2***	0.40	49.7 ± 9.6	45.8 ± 9.2**	0.44	51.5 ± 11.7	46.7 ± 7.3*	0.49
HbR (μM)	24.4 ± 3.4	28.1 ± 5.3**	0.85	24.4 ± 3.4	28.1 ± 5.3**	0.81	25.0 ± 2.4	28.0 ± 4.7*	0.76

Values are expressed as mean ± standard deviation. (N = 11) at baseline of the first session (Pre-Ex1), at the end of the first session (Ex1), at baseline of the second session (Pre-Ex2), at the end of the second session (Ex2), at baseline of the third (Pre-Ex3), and at the end of the third session (Ex3). Data were averaged over 15 s for each time point. ScO_2_, cerebral hemoglobin oxygen saturation; HbT, total hemoglobin concentration in the brain tissue; HBO, oxyhemoglobin concentration in the brain tissue; HHb, deoxyhemoglobin concentration in the brain tissue; ES(d), effect sizes (Cohen’s d). For Ex1, Ex2, and Ex3, these time points differed among ScO_2_, HbT, HbO, and HbR because data were demonstrated at time points when the largest changes were observed. For ScO_2_, values at 105, 15 and 30 s after exercise termination of Ex1, Ex2, and Ex3, respectively. For HbT, values at 0 s after exercise termination of all conditions. For HbO, values at 0 s for Ex1 and Ex2 and 30 s after exercise termination of Ex1 and Ex2,and at 30 s after exercise termination. For HbR, values at 120, 90, and 45 s after exercise termination of for Ex1, Ex2, and Ex3, respectively. Significant difference compared to Pre-Ex1 for Ex1, compared to Pre-Ex2 for Ex2, and compared to Pre-Ex3 for Ex3 *P < 0.05, **P < 0.01, ***P < 0.001.

### Warmup and 2,000-m All-Out Row

**FIGURE 4:**
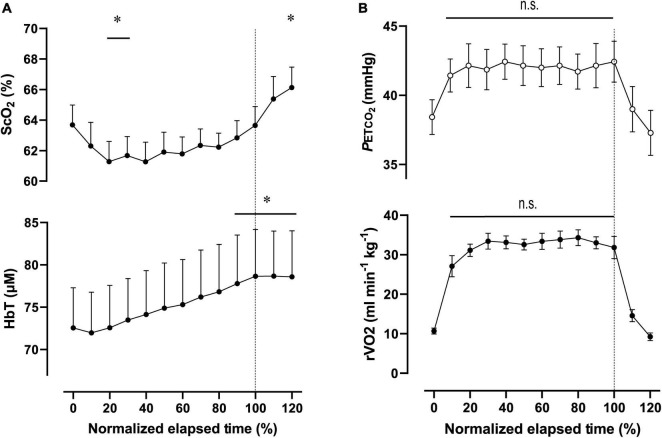
Cerebral oxygenation variables from near-infrared spectroscopy obtained in the prefrontal cortex **(A)** and respiratory variables **(B)** during the 2,000-m warm-up row (moderate-intensity exercise). Individual total elapsed times were normalized to the scale of 100% because of inter-individual differences. Solid lines represent the time point for 100% of total elapsed time (the end of exercise). ScO_2_, cerebral hemoglobin oxygen saturation; HbT, total hemoglobin concentration in the brain tissue; r$\dot{V}$O_2_, relative pulmonary oxygen consumption; P_*ET*_CO_2_, end-tidal carbon dioxide. Significant difference compared to baseline; *P < 0.05. Significant difference compared to 100% of total elapsed time (the end of exercise); *P* > 0.05 was not significant (n.s.). Statistical differences between each timepoint were determined using a one-way analysis of variance for repeated measures and Turkey’s *post hoc* multiple comparisons test.

Table 6 contains a summary of cardiovascular and respiratory variables at the baseline and at the end of ExM and Ex_2000_. A summary of NIRS variables during the session are shown in Table 7. Changes in ScO_2_ and [HbT] throughout the exercise session are presented with those in P_*ET*_CO_2_ and relative $\dot{V}$O_2_ in Figure 4. For both ExM and Ex_2000_, individual total elapsed times were normalized to the scale of 100% because inter-individual differences were apparent.

**TABLE 6:** Cardiovascular and ventilatory variables in response to 2,000 m moderate-intensity (warming up) followed by 2,000 m maximal rowing ergometer exercises.

	2,000 m moderate-intensity rowing		2,000 m maximal rowing	
	Rest	Ex terminated	Rest	Ex terminated
Heart rate (beats min^–1^)	92 ± 5	143 ± 15***	109 ± 13^†^	183 ± 16***
$\dot{V}$_*E*_ (breaths min^–1^)	22 ± 4	64 ± 5***	22 ± 4	144 ± 13***
R_*f*_ (breaths min^–1^)	21 ± 7	37 ± 4***	25 ± 5	62 ± 7***
$\dot{V}$O_2p*eak*_ (l min^–1^)	0.8 ± 0.1	2.0 ± 0.2***	0.7 ± 0.1	4.0 ± 0.6***
Relative $\dot{V}$O_2_ (ml min^–1^kg^–1^)	11 ± 2	34 ± 5***	10 ± 2	51 ± 7***
P_*ET*_CO_2_ (mmHg)	39 ± 2	43 ± 3***	38 ± 4	33 ± 2**
$\dot{V}$CO_2_ (l min^–1^)	0.8 ± 0.1	2.0 ± 0.2***	0.7 ± 0.1	4.0 ± 0.7***
RER	0.94 ± 0.12	0.95 ± 0.01	1.00 ± 0.16	1.10 ± 0.05*

Values are expressed as mean ± standard deviation. N = 7 at baseline (Rest) and at exercise termination (Ex terminated). Data were averaged over 15 s for each time point. $\dot{V}$_E_, minute ventilation; R_f_, breathing frequency;$\dot{V}$O_2peak_, pulmonary oxygen consumption; P_ET_CO_2_, end-tidal carbon dioxide,$\dot{V}$CO_2_, carbon dioxide production; RER, respiratory exchange ratio. Significant difference compared to Rest: *P < 0.05, **P < 0.01, ***P < 0.001. Significant difference compared to Rest of 2,000-m moderate-intensity rowing exercise: ^†^P < 0.01.

**TABLE 7:** Summary of NIRS data in response to 2,000 m moderate intensity (warming up) followed by 2,000 m maximal rowing ergometer exercises.

	2,000 m moderate-intensity rowing			
	Rest	100% of elapsed time	120% of elapsed time (post exercise)	ES (η^2^-partial)

ScO_2_ (%)	63.7 ± 3.5	63.6 ± 3.3	66.1 ± 3.5**,^†^	0.860
HbT (μM)	71.2 ± 13.1	76.6 ± 14.9**	76.4 ± 14.4**	0.825
HbO (μM)	44.7 ± 8.6	48.5 ± 9.8*	50.0 ± 10.6**	0.827
HbR (μM)	26.5 ± 4.9	28.1 ± 5.7*	26.4 ± 4.3^†^	0.668

	**2,000 m maximal rowing**			
	**Rest**	**100% of elapsed time**	**120% of elapsed time (post exercise)**	**ES (η^2^-partial)**

ScO_2_ (%)	64.5 ± 4.0	49.2 ± 3.3***	56.9 ± 3.0**,^††^	0.996
HbT (μM)	75.2 ± 13.5§	76.0 ± 15.9	83.2 ± 15.9**,^††^	0.951
HbO (μM)	48.7 ± 9.3	37.6 ± 8.0***	47.6 ± 9.8^††^	0.970
HbR (μM)	26.6 ± 4.9	38.6 ± 7.7***	35.7 ± 6.6**,^†^	0.942

Values are expressed as mean ± standard deviation. N = 7 at baseline (Rest), at 100% and at 120% of total elapsed time (post exercise). Individual total elapsed times were normalized to the scale of 100% because of inter-individual differences. Es, effect size; ScO_2_, cerebral hemoglobin oxygen saturation; HbT, total hemoglobin concentration in the brain tissue; HBO, oxyhemoglobin concentration in the brain tissue; HHb, deoxyhemoglobin concentration in the brain tissue. Significant difference compared to Rest: *P < 0.05, **P < 0.01, ***P < 0.001. Significant difference compared to 100% of total elapsed time: ^†^P < 0.05, ^††^P < 0.001. Significant difference compared to Rest in 2,000-m moderate-intensity rowing: ^§^ P < 0.05.

For ExM, PO was 127 ± 18 W during 564 ± 27 s, corresponding to 45 ± 7% MPO. At the end of ExM, the values of the cardiovascular and ventilatory variables (except for RER) increased. Relative $\dot{V}$O_2_ increased to 61 ± 5% of $\dot{V}$O_2p*eak*_. $\dot{V}$O_2p*eak*_ and P_*ET*_CO_2_ remained at the same level, but not during the initial phase. [ScO_2_] significantly decreased compared to that at rest to 61.3 ± 3.5% from 20 to 30% to the normalized scale; then, it continued to increase, attaining the highest value of 66.1 ± 3.5% at the end of the session. [HbT] gradually increased and attained the highest value of 78.7 ± 14.1 μM at 110% of the normalized scale. The magnitude of the changes was similar among NIRS variables.

**FIGURE 5:**
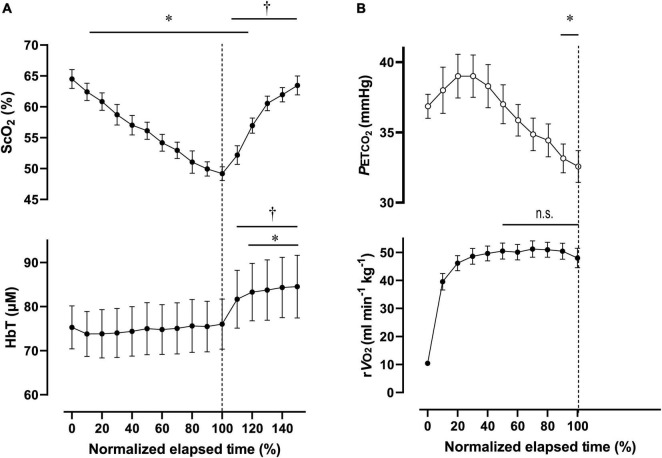
Cerebral oxygenation variables from near-infrared spectroscopy obtained in the prefrontal cortex **(A)** and respiratory variables **(B)** during the 2,000-m all-out row (maximal-intensity exercise). Individual total elapsed times were normalized to the scale of 100% because of inter-individual differences. Solid lines represent time point for 100% of total elapsed time (the end of exercise). ScO_2_, cerebral hemoglobin oxygen saturation; HbT, total hemoglobin concentration in the brain tissue; r$\dot{V}$O_2_, relative pulmonary oxygen consumption; P_*ET*_CO_2_, end-tidal carbon dioxide. Significant difference compared to baseline; *P < 0.05. *P* > 0.05 was not significant (n.s.). Significant difference compared to 100% of elapsed time (the end of exercise); ^†^*P* < 0.05. Statistical differences between each timepoint were determined using a one-way analysis of variance for repeated measures and Turkey’s *post hoc* multiple comparisons test.

For Ex_2000_, PO was 262 ± 39 W during 445 ± 26 s, corresponding to 91 ± 4% MPO. At the end of Ex_2000_, the cardiovascular and ventilatory variables except for RER and P_*ET*_CO_2_ increased. Relative $\dot{V}$O_2_ increased to 95 ± 9% of $\dot{V}$O_2p*eak*_. HR and P_*ET*_CO_2_ significantly increased at baseline during Ex_2000_ as a result of the warmup procedures, but the other variables were similar. Changes in [ScO_2_] and [HbT] throughout the session are presented with those of P_*ET*_CO_2_ and relative $\dot{V}$O_2_ in Figure 5. [ScO_2_] decreased immediately after the exercise started and reached its nadir of 49.2 ± 3.0% at the termination of exercise; then it began to increase to the same level as that at baseline at 130% of the normalized scale. [HbT] stayed at the same level during Ex_2000_ and began to increase after the termination of exercise, and significantly increased compared to the rest at 120% of the normalized scale thereafter. When comparing the baseline variables of ExM and Ex_2000_, only [HbT] had a significantly higher value. The magnitude of the changes in NIRS variables of Ex_2000_ was larger than that of ExM.

### Relative Changes in Cerebral Oxygenation and Estimation of rCBV and rCMRO_2_

#### At the End of Exercise

**FIGURE 6:**
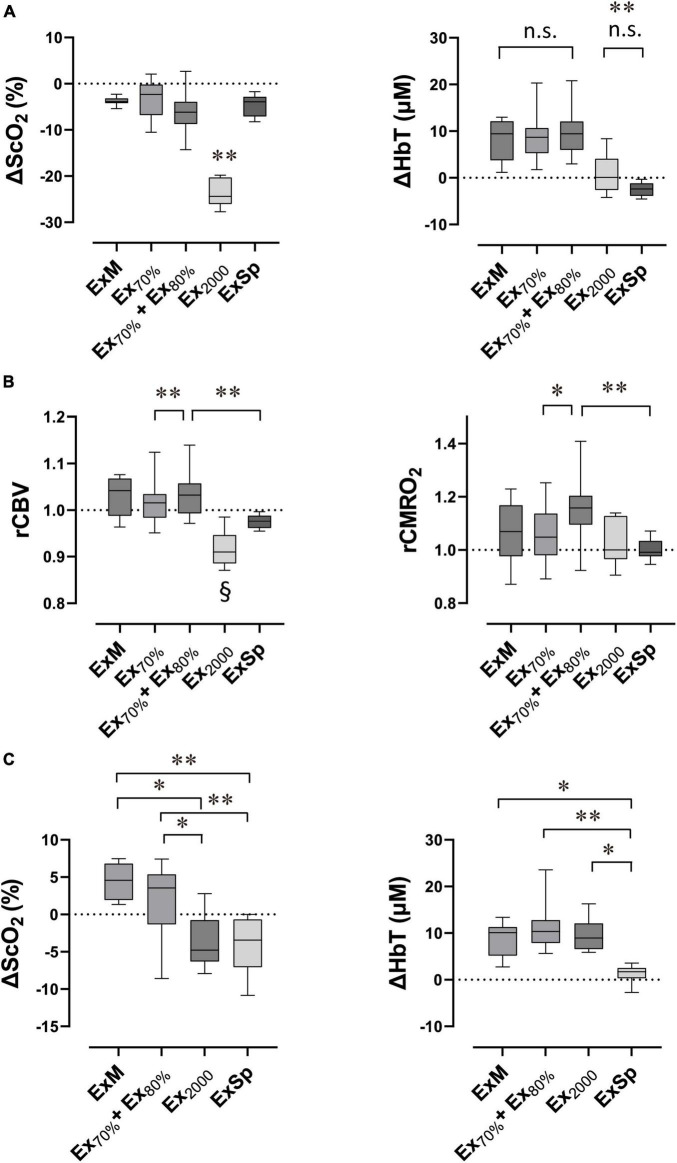
Box plots show the extent of changes in variables compared with the baseline in response to exercise performed with various intensities (*N* = 11 for Ex_70%_, Ex_70%_ + Ex_80%_ and ExSp; *N* = 7 for ExM and Ex_2000_). Lower and upper box boundaries represent the 25th and 75th percentiles, respectively. Lines inside the box represent the median. Lower and upper error lines represent 10th and 90th percentiles, respectively. Relative changes in cerebral oxygenation at the end of exercise **(A)**, estimated relative changes in rCBV and rCMRO_2_ at the end of exercise **(B),** and relative changes in cerebral oxygenation after exercise termination **(C)**. ΔScO_2_, relative changes in cerebral hemoglobin oxygen saturation; ΔHbT, relative changes in total hemoglobin concentration in the brain tissue; rCBV, estimated relative changes in cerebral blood flow; rCMRO_2_, estimated cerebral metabolic rates for oxygen; ExM, moderate-intensity exercise (2,000 m warmup row); Ex_70%_, exercise at 70% of $\dot{V}$O_2p*eak*_; E_70%_ + Ex_80%_, exercise at 70% of $\dot{V}$O_2p*eak*_ followed by exercise at 80% of $\dot{V}$O_2p*eak*_; Ex_2000_, maximal exercise (2,000 m all-out row); ExSp, three sessions of supramaximal intensity exercise. **(A)** ΔScO_2_ evoked by Ex_2000_ was significantly different from those evoked by the other four intensities (^**^P < 0.01), and ΔHbT evoked by Ex_2000_ and ExSp were significantly different from those evoked by the other three intensities (^**^P < 0.01). Variables were not significantly different between Ex_2000_ and ExSp or among ExM, Ex_70%_, and ExH_70%_ + ExH_80%_ (n.s. *P* > 0.05 was not significant [n.s.]). **(B,C)** rCBV evoked by Ex_2000_ was significantly different from those evoked by other four intensities (§*P* < 0.05). Pair-wised significant differences are indicated (*P < 0.05 and ^**^P < 0.01). Statistical differences between variables were analyzed with a one-way analysis of variance and Turkey’s *post hoc* multiple comparisons test.

The ΔScO_2_ and ΔHbT (Figure 6A) and estimated rCBV and rCMRO_2_ (Figure 6B), comparing baseline values with values at the end of the five intensities of rowing exercise are presented in Figure 6. Because there were no significant differences among ΔScO_2_ [*F*(1.87, 18.67) = 0.35; *p* = 0.70] and ΔHbT [*F*(1.91, 19.10) = 0.18; *p* = 0.83] at the end of three sessions of ExSp or for rCBV and rCMRO2, these values obtained during the first session were used as the effect of ExSp. The ΔScO_2_ was −23.0 ± 2.9% at the end of Ex_2000_, and it was significantly greater than all the other intensities; however, the ΔScO_2_ were similar among ExM, Ex_70%_, Ex_70%_ + Ex_80%_, and ExSp. The ΔHbT were 8.3 ± 4.6, 8.8 ± 4.8, and 9.7 ± 4.8% during ExM, Ex_70%_ and Ex_70%_ + Ex_80%_, respectively. The ΔHbT at the end of ExM, Ex_70%_, and Ex_70%_ + Ex_80%_ were significantly greater than those of Ex_2000_ and ExSp. The estimated rCBV values at the end of each exercise intensity were 1.03 ± 0.04, 1.02 ± 0.05, 1.03 ± 0.04, 0.92 ± 0.04, and 0.98 ± 0.02 for ExM, Ex_70%_, Ex_70%_ + Ex_80%_, Ex_2000_, and ExSp, respectively. For Ex_2000_, the rCBV was significantly lower than that of the other intensities. The rCBV of Ex_70%_ + Ex_80%_ was significantly higher than that of Ex_70%_ and ExSp. The estimated rCMRO_2_ values at the end of each exercise intensity were 1.06 ± 0.12, 1.06 ± 0.12, 1.17 ± 0.14, 1.02 ± 0.09, and 1.00 ± 0.04 for ExM, Ex_70%_, Ex_70%_ + Ex_80%_, Ex_2000_, and ExSp, respectively. For Ex_70%_ + Ex_80%_, the rCMRO_2_ values were significantly higher than those of Ex_70%_, and ExSp.

#### After Termination of Exercise

The ΔScO_2_ and ΔHbT observed after four sessions of different exercise intensities are presented in Figure 6C. Because there were not significant differences among ΔScO_2_ [*F*(1.84, 18.40) = 2.42; *p* = 0.12], ΔHbT [*F*(1.26, 12.53) = 0.75; *p* = 0.43] after three sessions of ExSp, we used these values obtained after the first session of ExSp. The ΔScO_2_ were 4.3 ± 2.3, 2.1 ± 4.5, −3.7 ± 3.7, and −4.3 ± 3.5% after ExM, Ex_70%_ + Ex_80%_, Ex_2000_, and ExSp, respectively. The ΔScO_2_ after ExM and Ex_70%_ + Ex_80%_ were significantly greater than those after Ex_2000_ and ExSp. ΔHbT were 8.4 ± 3.9, 11.5 ± 5.2, 9.6 ± 3.8, and 1.3 ± 1.8% after ExM, Ex_70%_ + Ex_80%_, Ex_2000_, and ExSp, respectively. The ΔHbT was significantly less after ExSp than after all other intensities.

#### Effect of Oxidative Capacity

When the ScO_2_ and [HbT] significantly changed compared to rest, the ΔScO_2_ correlated with $\dot{V}$O_2p*eak*_ at the end of Ex_2000_ (*r* = −0.86; *p* = 0.01). For other exercise intensities, the ΔScO_2_ and ΔHbT were not correlated with MPO nor with $\dot{V}$O_2p*eak*_ (Supplementary Tables 1, 2).

## Discussion

There were several main findings of this study that investigated cerebral oxygenation in the PFC during various types of exercise intensities during rowing and examined the association between altered sensations and cerebral oxygenation during exercise at 70 and 80% of $\dot{V}$O_2p*eak*_. First, quantitative measurements using TRS demonstrated a significant decrease in [ScO_2_], but not in [HbT] in accordance with the alteration of the sense of effort (indexed from RPE) when a higher intensity of exercise was added during 13 min of dual-stage constant-load rowing exercise consisting of 67 and 79% $\dot{V}$O_2p*eak*_. Second, the 13 min of dual-stage constant-load rowing exercise induced a decrease in the negative affect of mood status (indexed by PANAS) accompanied by increases in both [ScO_2_] and [HbT] in the PFC after exercise termination. Third, exhaustion evoked by the 2,000-m all-out row induced a distinct decrease of 23% in ScO_2_, whereas exhaustion evoked by the 2,000-m all-out row and 1-min of ExSp induced declines in [HbT]. Fourth, exercise-induced changes in ScO_2_ correlated with $\dot{V}$O_2p*eak*_ at the 2,000-m all-out row. Finally, the estimated rCBV and rCMRO_2_ derived from quantitative values obtained by TRS demonstrated how they changed in accordance with exercise intensity.

### Alterations in the Sensation of Effort and Cerebral Oxygenation Induced by High-Intensity Rowing

During the dual-stage constant-load rowing exercise at Ex_70%_ and Ex_80%_, the RPE changed from 12 to 13. In accordance with this alteration in the sensation of effort, accompanied by distinct changes in HR (156–167 bpm), CO (15.0–18.1 L min^–1^), MBP (101–109 mmHg), and respiratory variables, ScO_2_ significantly decreased from 62.0 ± 3.8 to 60.2 ± 4.0%. As exercise intensity is determined by physiological variables, with HR being most used for a typical exercise prescription, oxygenation in the PFC detects changes in exercise intensity during rowing. Because the medial PFC is involved in both executive control and the emotional salience network in conjunction with many other brain regions, such as the insular and anterior cingulate cortex, oxygenation in the PFC would be a surrogate for observing interoception caused by rowing exercise (Craig, 2009). Additionally, the PFC has been explored in NIRS studies that investigated the underlying mechanism of the pacing strategy related to an experimental model of time-to-exhaustion (De Wachter et al., 2021). From the perspective of the regulation of endurance exercise in the brain, the PFC has a key role in brain regions such as the anterior cingulate cortex and premotor area cortex, which integrate afferent and efferent information mandatory for executive function for endurance exercise (Robertson and Marino, 2016).

The finding that [HbT] did not change between Ex_70%_ and Ex_80%_ but did increase from the baseline by 8.6 and 10.0%, respectively, demonstrated the CBF stability. The combination of ScO_2_ and [HbT] estimated the difference in rCMRO_2_ between Ex_70%_ and Ex_80%_ (Figure 6). Increased CMRO_2_ with Ex_80%_ despite unchanged CBF because of higher-intensity exercise would indicate fluctuation in neurovascular coupling, during which an increase in the metabolic demand of neurons during activity would induce a further increase in the CBF (Attwell et al., 2010). A previous study using NIRS demonstrated a cerebral oxygenation threshold of 87% for $\dot{V}$O_2*max*_, corresponding to VT2, whereby oxygenation in the PFC decreased during the incremental exercise test to exhaustion (Rupp and Perrey, 2008). During our study, the finding that ScO_2_ decreased as [HbT] stayed at the same level when intensity changed from Ex_70%_ to Ex_80%_ (from 67 to 79% of $\dot{V}$O_2p*eak*_) seemed compatible with the oxygenation threshold advocated by the previous study. We speculated that this difference in the threshold would be attributable to the study population and protocol.

After the combined sessions of Ex_70%_ and Ex_80%_, both ScO_2_ and [HbT] significantly increased 3 min after exercise termination compared with the baseline. [HbT] notably increased by 12.1%, where ScO_2_ increased by 3.3% (Table 3 and Figure 6). We speculated that this increase in [HbT] would allow an increase in CBF, thus surpassing the change in HGB. A reason for the possible increase in CBF is that a significant increase in CO was identified 3 min after exercise termination. Since a single session of exercise improves mood and reduces subjective symptoms of anxiety in healthy non-anxious adults (Petruzzello et al., 1991; Smith, 2013), we measured the alterations in the mood status before and after the dual-stage constant-load rowing exercise to identify whether these alterations might be reflected in the oxygenation in the PFC. There was a significant decrease in the negative PANAS scores, but the positive scores remained at the same level. In addition to the possible increase in CBF, this small increase in ScO_2_ could be linked to an increase in CMRO_2_. Accordingly, it was speculated that these positive changes in CBF and CMRO_2_ in the PFC might be possibly associated with alterations in mood. Although the results of our study could not identify neurophysiological mechanisms of the beneficial effects on mood that are evoked by exercise, these changes might trigger consecutive procedures in the network existing in the large regions of the brain that are associated with the development of post-exercise antidepressive effects.

### Exhaustion Evoked by Maximal Rowing and Cerebral Oxygenation

With exhaustion caused by the 2,000 m all-out row, the SaO_2_ may decrease below 85% (Nielsen et al., 1998). This systemic deterioration in oxygen delivery is critical for ScO_2_ (Nielsen et al., 1999). Our results are in line with those of previous studies that investigated maximal exercise to exhaustion. However, the extent of decrease of 23% in ScO_2_ was greater than that reported by previous studies (17%) (Nielsen et al., 1999). Although we could not determine actual changes in the CBF, the estimated rCMRO_2_ was retained as the baseline value because of the balance between systemic arterial desaturation and decreased ScO_2_.

To evaluate another type of maximal effort to develop exhaustion, we included ExSp. In contrast to other exercise intensities, during ExSp, [HbT] decreased immediately after exercise was started. Because the duration and intensity of ExSp (1 min at 130 or 140% of $\dot{V}$O_2p*eak*_) were different from those of other exercise sessions, the extent of changes in ScO_2_ and in [HbT] were smaller than those during other sessions. When considering the three sessions of ExSp as a block, [HbT] tended to increase as the sessions were repeated. Additionally, in contrast to HbT, P_*ET*_CO_2_ declined with repetitive sessions at supramaximal intensity. CO_2_ has an influences on regional CBF and CBV (Ito et al., 2003), and a decrease of 6 mmHg from the baseline value of the first session to that of third session in ExSp would induce a decreased of 7.8% in the CBV. Although, [HbT] did not change between the baseline of the first and the third bout, CBV may decrease by 9% because of the approximately 10% increase in HGB, similar to that observed with Ex_2000_. Accordingly, this finding may indicate that the cerebrovascular response to CO_2_ is applicable to these intermittent repetitive exercise sessions.

During Ex_2000_ and ExSp, with increasing intensity or an accumulated load, a severe sensation occurred, but the rowers endured it with exertion. Thereafter, PO decreased with fatigue and, eventually, stop exercising. During the present study, these phenomena at exhaustion were interpreted as peripheral fatigue (Noakes et al., 2005), which is explained by a catastrophic failure of homeostasis leading to skeletal muscle dysfunctions, and also as central fatigue, which refers to all the processes implicated in motoneuronal activation that can be modulated at the spinal and/or supraspinal levels (Gandevia, 2001). Central fatigue is a theoretical explanation of where afferent information of the body is integrated into brain areas, networks, and efferent pathway to stop movement. According to NIRS and functional imaging, such as PET and functional magnetic resonance imaging studies, the PFC may be intimately involved in the capacity to tolerate high levels of physical exertion and possibly in the determination of exercise termination (Robertson and Marino, 2016).

### Effects of Heterogeneous Characteristics of Rowers

To identify changes in cerebral oxygenation, we investigated all participants as a group despite the heterogeneous characteristics. Because the participants of this study were consisted of two groups of six competitive and five recreational rowers, there was a large difference of $\dot{V}$O_2p*eak*_ (from 43 to 65 mL min^–1^kg^–1^) among the participants. However, as the groups were not equally balanced regarding sex and age, we did not analyze the data by comparing the two groups. Accordingly, we analyzed whether ΔScO_2_ and ΔHbT were associate $\dot{V}$O_2p*eak*_. To evaluate Ex_2000_, we investigated rowers only if they tolerated maximal exercise. Consequently, our observations seem to be limited to cases of true exhaustion. If the maximum sensation of effort was attained among rowers at the end of Ex_2000_, then the oxidative capacity was correlated with ΔScO_2._ Because we investigated only seven rowers, further investigations are required to determine whether the oxidative capacity affects changes in oxygenation in the PFC.

### Estimation of Cerebral Metabolic Rate for Oxygen Resulting From Rowing Exercise

At exhaustion evoked by maximal exercise, the decrease in ScO_2_ oxygenation was likely caused by the decreased regional CBF combined with the increased CMRO_2_ (Nielsen et al., 1999; González-Alonso et al., 2004). To clarify this uncoupling between the oxygen supply and demand, we attempted to estimate CMRO_2_. However, we could not explore the absolute values of CMRO_2_ because the CBF could not be determined by CBV, which is assessed by TRS. An advantage of TRS is that theoretically quantitative measures of [HbT] can be converted to CBV using the molecular weight of hemoglobin (64,500 g/Mol) and brain tissue density (1.05 g/mL) if the individual HGB was measured during exercise. A previous study applied simultaneous measurements of TRS and PET and demonstrated that a good correlation coefficient was obtained between TRS-derived CBV and PET-derived CBV, and that the absolute CBV levels found with TRS were lower than those found with PET (Ohmae et al., 2006). Given that CBV could be correctly assessed by TRS, and that arterial and blood sampling could determine SaO_2_, which was substituted by the values reported during this study (Nielsen et al., 1998; Nielsen, 2003; González-Alonso et al., 2004), CMRO_2_ was not available if the association between CBF and CBV was not correct. Using PET, the association between the CBF and CBV has been investigated, but not sufficiently (Grubb et al., 1974). During a previous PET study, changes in the CBF during hypercapnia and hypocapnia were greater than those in CBV. Therefore, the CBV and CBF might change in a common direction, at least as a result of physiological stimulation.

### Limitations

It is worth mentioning that small sample size, possible selection bias, and several methodological limitations in this study do not allow to draw generalizable conclusions. Limitations included the lack of a measurements of individual blood lactate during the incremental exercise test that could clearly define the boundaries of exercise intensities by the first lactate threshold (LT1) and second lactate threshold (LT2). Because we used 70 and 80% of $\dot{V}$O_2p*eak*_ as Ex_70%_ and Ex_80%_ and referred to VT1 and VT2, it is not impossible to interpret that these two intensity domains during this study correspond to above LT1 and between LT1 and LT2, respectively. Because of the heterogeneous background of the participants, 70% of $\dot{V}$O_2p*eak*_ was slightly lower than the VT1 for the recreational rowers, while 80% of $\dot{V}$O_2p*eak*_ was lower than the VT2 for all of the participants. Caution is warranted when interpreting the discrimination of Ex_70%_ and Ex_80%_, because inter-subject variance in ScO_2_ was large in response to the exercise of the same intensity. Additionally, because the present study used the impedance cardiograph (Physio Flow) to measure CO during rowing exercise in Ex_70%_ and Ex_80%_ rowing exercise, CO seemed low considering a near-linear relationship with an approximately 6.1 slope between CO and $\dot{V}$O_2_ (Astrand et al., 1964). However, our results are compatible with those of a recent study of stroke volume (SV) measured using a pulse contour analysis to determine waveform of arterial pressure during constant-load rowing exercise corresponding to 130 and 160 beats min^–1^ (Sejersen et al., 2021). This underestimation would be attributable to physiological characteristics of submaximal-intensity rowing which induces large fluctuations in SV while breathing similar to that occurring with a Valsalva-like maneuver, using a rowing cycle (24–26 min^–1^ during the present study) (Sejersen et al., 2021). Consequently, because prompt changes in central venous pressure and arterial blood pressure would induce rapid oscillation of systemic vascular resistance, CO might be underestimated by 5 s using impedance cardiography. The low fitness levels of athletes involved in the present study were considered and did not indicate that similar results would be obtained for highly trained athletes. The NIRS device was limited by artifacts caused by head movement and sweat during prolonged exercise, especially in high-intensity exercise. We carefully observed and removed errors caused by artifacts; consequently, NIRS data were observed in the right PFC of only two rowers. Because the physiological interpretation of the laterality of NIRS variables during a similar protocol of exercise has not been reported, further studies are required to examine the laterality effect. To detect the precise location in PFC, additional research involving magnetic resonance imaging should be performed to clarify the optimal area for NIRS data collection. Another limitation was that arterial blood sampling was not performed to measure HGB and SaO_2_, which is mandatory to detect CBV and calculate CMRO_2_ using the estimated CBF as well. Hence, further research including arterial blood sampling may validate and complement our results. Finally, regarding the accuracy of the determination of the association between CBF and CBV, additional studies involving simultaneous measurements of cerebral hemodynamics with PET and TRS are needed.

In summary, alterations in the sense of effort were paralleled by changes in PFC oxygenation, and positive changes in mood status were associated with cerebral perfusion and oxygen metabolism estimated by TRS. At exhaustion, with a possible decrease in CBF, a decrease in ScO_2_ could be attributed to the maintenance of rCMRO_2_. Oxidative capacity of rowers correlated with changes in ScO_2_ when exhaustion was evoked by the 2,000-m all-out row. TRS potentially measures CMRO_2_ if the correct association between CBV and CBF is determined by further multidisciplinary investigations. As recent reviews suggested that aspects of CBF and oxygen metabolism at exhaustive rowing remain unresolved (Volianitis et al., 2020; Treff et al., 2021), real-time measures of cerebral perfusion and metabolism during high-intensity and maximal rowing should be obtainable with future developments.

## Data Availability Statement

The original contributions presented in the study are included in the article/Supplementary Material, further inquiries can be directed to the corresponding author/s.

## Ethics Statement

The studies involving human participants were reviewed and approved by the Ethics Committee of the Faculty of Sociology, Aomori University. The patients/participants provided their written informed consent to participate in this study.

## Author Contributions

MH designed this study based on the previous researches collaborated with KT and YS. KT prepared physiological measurements. HS and AF prepared and evaluated psychological assessment. MH performed the research and data collection. MH and YS analyzed and interpreted the data. MH wrote the manuscript with the assistance of YS and YK. YK supervised the whole program. All authors critically reviewed and approved the manuscript prior to submission.

## Conflict of Interest

The authors declare that the research was conducted in the absence of any commercial or financial relationships that could be construed as a potential conflict of interest.

## Publisher’s Note

All claims expressed in this article are solely those of the authors and do not necessarily represent those of their affiliated organizations, or those of the publisher, the editors and the reviewers. Any product that may be evaluated in this article, or claim that may be made by its manufacturer, is not guaranteed or endorsed by the publisher.
