# Supplementary material for: Estimation of Cerebral Hemodynamics and Oxygenation During Various Intensities of Rowing Exercise: An NIRS Study
Source: Front Physiol. 2022 Mar 2;13:828357. doi: 10.3389/fphys.2022.828357 (PMC8924415; doi:10.3389/fphys.2022.828357)
Supplement: Supplementary file 1 [file Table_1.docx]

Table S1. Correlations between relative changes in NIRS variables and exercise capacity at termination of exercise sessions.

|  | $\dot{V}$O_2peak_ (l min^-1^) | | Relative $\dot{V}$O_2peak_ (ml min^-1^ kg^-1^) | | MPO (watt) | |
| --- | --- | --- | --- | --- | --- | --- |
|  | r coefficient | P value | r coefficient | P value | r coefficient | P value |
| ΔScO_2_ (n =11) |  |  |  |  |  |  |
| Ex_70%_ + Ex_80%_ | -0.52 | 0.10 | -0.10 | 0.76 | -0.54 | 0.08 |
| ExSp | -0.30 | 0.37 | -0.26 | 0.44 | -0.22 | 0.52 |
| ΔScO_2_ (n= 7) |  |  |  |  |  |  |
| Ex_2000_ | -0.86 | 0.01 | -0.70 | 0.08 | -0.74 | 0.06 |
| ΔHbT (n =11) |  |  |  |  |  |  |
| Ex_70%_ | 0.16 | 0.63 | 0.01 | 0.98 | 0.12 | 0.74 |
| Ex_70%_ + Ex_80%_ | 0.20 | 0.54 | -0.03 | 0.92 | 0.18 | 0.58 |
| ΔHbT (n =7) |  |  |  |  |  |  |
| ExM | 0.42 | 0.35 | 0.27 | 0.56 | 0.04 | 0.93 |

Correlations were analyzed when significant changes in ScO_2_ or [HbT] for any exercise session were identified.

$\dot{V}$O_2peak_, peak pulmonary oxygen consumption: MPO, maximal power output obtained during the rowing ergometer incremental test; ΔScO_2_, relative changes in cerebral hemoglobin oxygen saturation; ΔHbT, relative changes in total hemoglobin;; ExM, moderate-intensity exercise (2000m warmup row); Ex_70%_, exercise at 70% of $\dot{V}$O_2peak_, Ex_70%_ + Ex_80%_, exercise at 70% of $\dot{V}$O_2peak_ followed by exercise at 80% of $\dot{V}$O_2peak_; Ex2000, maximal exercise (2000m all-out row); ExSp, three bouts of supramaximal intensity exercise.

Table S2. Correlations between relative changes in NIRS variables and exercise capacity after termination of exercise sessions.

|  | $\dot{V}$O_2peak_ (l min^-1^) | | Relative $\dot{V}$O_2peak_ (ml min^-1^ kg^-1^) | | MPO (watt) | |
| --- | --- | --- | --- | --- | --- | --- |
|  | r coefficient | P value | r coefficient | P value | r coefficient | P value |
| ΔScO_2_ (n =11) |  |  |  |  |  |  |
| ExSp | 0.18 | 0.60 | 0.08 | 0.81 | 0.27 | 0.42 |
| ΔScO_2_ (n= 7) |  |  |  |  |  |  |
| ExM | 0.35 | 0.44 | 0.41 | 0.36 | 0.06 | 0.90 |
| ΔHbT (n =11) |  |  |  |  |  |  |
| Ex_70%_ + Ex_80%_ | 0.40 | 0.22 | 0.08 | 0.83 | 0.37 | 0.26 |
| ΔHbT (n =7) |  |  |  |  |  |  |
| ExM | 0.58 | 0.17 | 0.22 | 0.63 | 0.27 | 0.56 |
| Ex_2000_ | -0.55 | 0.26 | -0.13 | 0.81 | -0.70 | 0.12 |

Correlations were analyzed when significant changes in ScO_2_ or [HbT] for any exercise session were identified.

$\dot{V}$O_2peak_, peak pulmonary oxygen consumption: MPO, maximal power output obtained during the rowing ergometer incremental test; ΔScO_2_, relative changes in cerebral hemoglobin oxygen saturation; ΔHbT, relative changes in total hemoglobin;; ExM, moderate-intensity exercise (2000m warmup row); Ex_70%_, exercise at 70% of $\dot{V}$O_2peak_, Ex_70%_ + Ex_80%_, exercise at 70% of $\dot{V}$O_2peak_ followed by exercise at 80% of $\dot{V}$O_2peak_; Ex2000, maximal exercise (2000m all-out row); ExSp, three bouts of supramaximal intensity exercise.
